# Development of microsatellites markers for the deep coral *Madracis myriaster* (Pocilloporidae: Anthozoa)

**DOI:** 10.1038/s41598-022-14322-7

**Published:** 2022-08-01

**Authors:** Diana C. Ballesteros-Contreras, Lina M. Barrios, Richard Preziosi

**Affiliations:** 1 Faculty of Science and Engineering, Manchester Metropolitan University-MMU, Manchester, UK; 2 Instituto de Investigaciones Marinas y Costeras de Colombia-INVEMAR, Santa Marta D.T.C.H., Santa Marta, Colombia; 3 School of Biological and Marine Sciences, The University of Plymouth, Plymouth, UK

**Keywords:** Biological techniques, Molecular biology, Ecology, Ocean sciences

## Abstract

In 2013 Colombia made an important step towards the construction and management of Marine Protected Areas (MPAs) by establishing the first Deep Corals National Park (PNNCP). Inside this MPA, the coral *Madracis myriaster* (Cnidaria: Pocilloporidae) was found as the main reef builder, offering habitat for many species of fish and invertebrates. In order to improve the study of deep-sea coral habitats, their connectivity and prospective management, nine new genetic markers (microsatellites) were developed for *M. myriaster* and tested in samples from PNNCP. We present the assessment of these markers, with a specificity for the deep coral, and its prospective use in future analysis for the PNNCP and other areas in the Caribbean and the Atlantic, where *M. myriaster* is reported. We also include an additional taxonomic analysis performed on samples of *M. myriaster* using scanning electron microscopy.

## Introduction

Deep-sea colonial corals are characterised by their three-dimensional structures, which harbour high biodiversity, supporting an extensive variety of organisms that are sources of food and economic wellbeing for local human populations^1–3^. These habitats are found on continental shelves, slopes, canyons, and seamounts between 150 and 1500 m depth^4^, clinging to small solid surfaces in the presence of currents, which promote the larvae and gametes dispersing, as well as accessibility to suspended food, given the absence of symbiont zooxanthellae^5^. The exploration of these habitats in the last decades has shown that deep-sea corals have undergone overexploitation of resources for economic purposes, due mainly to oil extraction and trawling^6,7^. Moreover, the combination of overfishing and ocean acidification, one of the side effects of climate change, is having direct consequences on the growth and health of marine organisms^8,9^, in particular deep-sea corals^10,11^.

The searching of deep-sea habitats in Colombia began in the 1970s^12^. As a result, between 1995 and 2012 the Colombian Institute for Marine and Coastal Research (INVEMAR), in collaboration with International and National institutions, focused their examination on one of the most diverse areas discovered, which was declared in 2013 as the “Deep Corals National Natural Park” (PNNCP by its abbreviation in Spanish). The PNNCP was the first marine protected area (MPA) with deep-sea corals in the Colombian Caribbean, bringing new information about the geomorphology, bathymetry and marine diversity of the area^12^. The PNNCP area is dominated by the coral *Madracis myriaster*, found between 120 and 350 m depth, although the distribution of the species in the Tropical Western Atlantic has been reported reaching 1220 m depth^2^. *M. myriaster* is an azooxanthellate coral from the Pocilloporidae family that shows a wide variety of morphologic growth patterns^13^, making its morphological identification and differentiation from its sister species *M. brueggemanni* difficult, when following the identification guides developed by Reyes et al*.* (2010). In addition, previous reports of *M. myriaster* suffered from a longstanding confusion with the shallow coral *M. auretenra*, previously known as *M. mirabilis*^14^.

Despite the development of new tools to explore these habitats around the world, there is still a lack of information about the distribution, genetic diversity and connectivity of deep-sea coral habitats^15^. The information regarding dispersion patterns around these habitats is necessary for the design and management of MPAs^16,17^, which is essential for conservation strategies^16^, promoting the production of eggs and larvae inside of the MPAs^11,15,18^ by physical delimitations and protecting the movement of individuals into adjacent areas.

In order to understand the gene flow among coral populations, genetic markers such as microsatellites^19,20^, have been used to estimate the dispersion of organisms by examining genetic differentiation of populations^21^, providing clear results for ecology and conservation studies^22^ in shallow corals^23–25^ and deep corals such as *Lophelia pertusa*^26^*, Goniocella dumosa, Madrepora oculata, and Solenosmilia variabilis*^27^ and others^28–30^. The aim of this study was to develop informative microsatellite markers for the species *M*. *myriaster*, with samples from the PNNCP, as a tool that will allow future understanding of the genetic structure of *M*. *myriaster* in the Colombian Caribbean and its gene flow among other deep reefs in the Caribbean Sea.

## Results

### Analysis of *Madracis myriaster* samples

**Figure 1:**
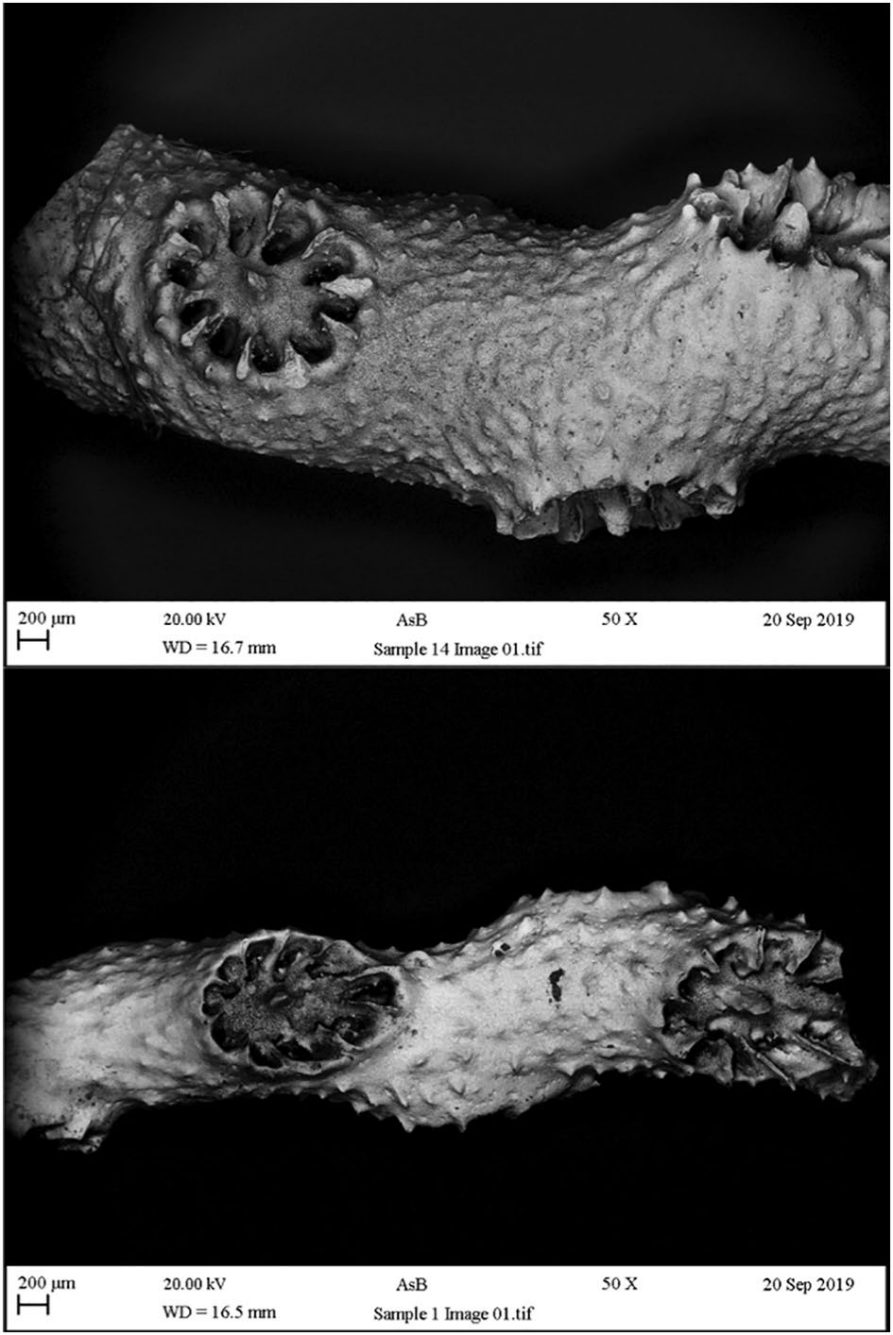
Scanning electron microscopy-SEM in samples from PNNCP*.* Top: general view sample identified as *M. myriaster*. Bottom: general view sample identify as *M.* cf. *myriaster.*

**Figure 2:**
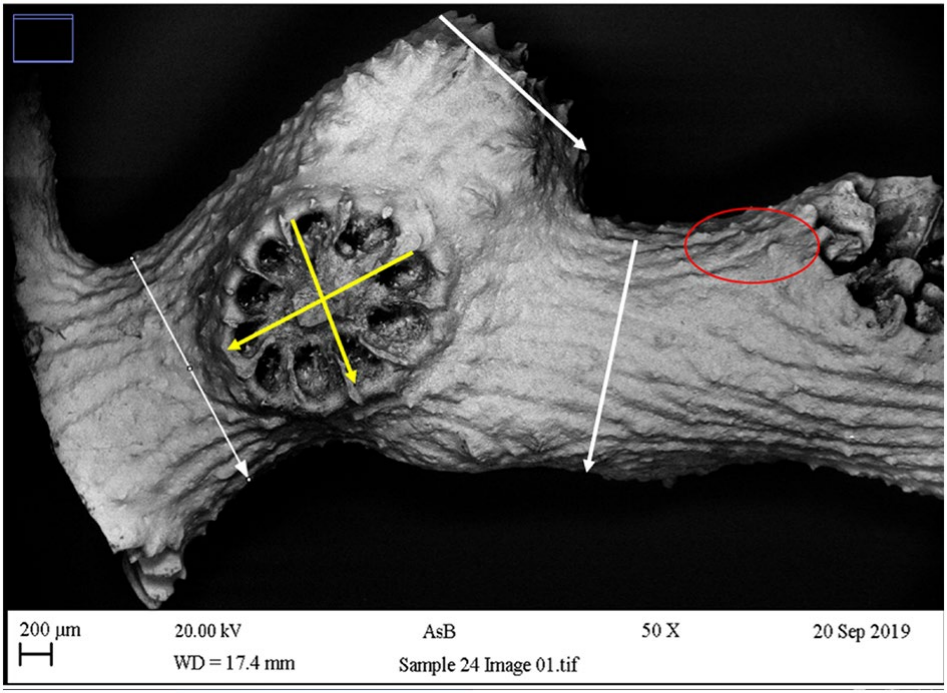
Example of scanning electronic microscopy-SEM analysis for the genus *Madracis* and measurements (program Image J) used for taxonomic identification. White lines correspond to measurements of the branches. Yellow lines are the measurements of the corallite. The red circle shows the presence of spines in the coral.

Analyses from the scanning electron microscopy-SEM, and statistical testing by t-test of the 26 samples of *M. myriaster* and *M.*cf*. myriaster * collected (Fig. 1), did not show a significant difference between the groups, either on the branch thickness of the samples (t-test = 0.45, df = 23) or the corallite diameter (t-test = 0.27, df = 23). The presence of spines in the samples did not show differentiation between groups and was not related to any group (Fig. 2). The analyses confirmed the suitability of the samples for the development of the microsatellites.

### PCR standardization for microsatellites and DNA sequencing

The test of the 14 microsatellite loci obtained from the filtering process (see methods) with the 26 M*. myriaster* samples showed correct amplification in 13 samples. The other 13 samples had clear electrophoresis bands, but unclear reading of the fragment sizes in “Fragman” and poor binning in ‘MsatAllele’ (Table 1). A total of nine microsatellite loci were confirmed for the deep-sea coral *M. myriaster*, using the analysis in “Poppr” and the criteria of 20% of missing data across the 13 samples. The primer specificity test, using samples from *M. auretenra, Montipora* sp. and *Antillogorgia* sp. under the same amplification conditions used for *M. myriaster*, confirmed no amplification on the other species.

**Table 1:** Locus information for the primers of *M. myriaster* with best quality of amplification.

Locus name	Primer sequences (5ʹ–3ʹ)	Repeat motif	Locus size range	Na	Ho	He	GenBank accession no
MMYR 1	F: CGCTGAGTTGAATCCTTGAAGGGC	AATG (44)	200–468	9	0.57	0.66	MT192036.1
	R: TTATCGATATCATCATTTCTCCCCGC						
MMYR 3	F: CAAACCAACGATAGAATAAACCAGCG	ACCG (24)	154–304	7	0.34	0.71	MT192037.1
	R: ATTCCAGCGGTCAGTTCAGTACCC						
MMYR 4	F: CCTTACGTGAAGGTGTCTTTGCG	AAAT (36)	146–466	11	0.53	0.87	MT192038.1
	R: TTAGAATATTGCTGACAAGGCCCGC						
MMYR 5	F: TGGTTGAGAAATTTCCGGTAGTCGC	AATC (72)	210–282	8	0.5	0.88	MT192039.1
	R: TTCTTCATGAGGGTGCTTTCCG						
MMYR 13	F: GTTCCAGAAAATATTCATGTCCCC	AAAC (36)	162–210	7	0.36	0.85	MT192040.1
	R: GGGTCCTTGGAACTTTGAGGG						
MMYR 26	F: GTGTGTCAAACTCGTCCTCCTGCG	TCCG (28)	274–370	6	0.38	0.82	MT192041.1
	R: CCGGCGGTTAGTAGTTTCTGCG						
MMYR 27	F: AAACACGCACTGGCCTATATGACCC	AAAC (44)	320–392	4	0.81	0.71	MT192042.1
	R: GTGCTTCCTTTGAATGAGAAAGCCG						
MMYR 30	F: CCCTTCCGAATTCAATGCTACGC	AAC (39)	290–410	6	0.64	0.77	MT192043.1
	R: CGTAGGACCAGTAACACCTCTCCGC						
MMYR 34	F: TATTCCTTGACAGCAGCATAGCGCC	TTC (33)	264–309	8	0.34	0.88	MT192044.1
	R: GTCATCAGCGCAAACACGAATCCC						

Na: Allele number per locus. Ho: Observed heterozygosity. He: Expected heterozygosity.

### Data analysis

The assessment of the genotyping results, performed with the final 26 DNA selected samples, showed the potential of these loci for further population genetics analyses. The test for clones, based on multilocus genotypes (‘Poppr’), did not find clones in the samples used. The allele number per locus (Na) was between 4 and 11. The observed and expected heterozygosity ranged from 0.334 to 0.81 and 0.66 to 0.88, respectively (Table 1). The *M. myriaster* total DNA sequencing information from Illumina MiSeq sequencing was uploaded to the SRA database (accession ID: **PRJNA611988).** Primer sequences for the nine confirmed microsatellite loci are available (Table 2) to facilitate future use in research.

**Table 2:** Information of 26 coral samples (*Madracis myriaster* complex) from the Cnidarian collection at MHNM.

	Catalogue Number	Project	Area	Station Code	Coordinates	Collection Date
1	336	Macrofauna I	PNN Tayrona	E8	11° 23ʹ 6.60ʺ N–74° 12ʹ 3.60ʺ W	03/10/1998
2	2012^a^	Macrofauna II	PNNCP	E155	9° 47ʹ .00ʺ N–76° 13ʹ 45.00ʺ W	26/03/2001
3	2013^a^	Macrofauna II	PNNCP	E155	9° 47ʹ 12.00ʺ N–76° 13ʹ 45.00ʺ W	26/03/2001
4	2013	Macrofauna II	PNNCP	E155	9° 47ʹ 12.00ʺ N–76° 13ʹ 45.00ʺ W	26/03/2001
5	2014^a^	Macrofauna II	PNNCP	E156	9° 47ʹ 1.00ʺ N–76° 14ʹ 12.00ʺ W	26/03/2001
6	2014	Macrofauna II	PNNCP	E156	9° 47ʹ 1.00ʺ N–76° 14ʹ 12.00ʺ W	26/03/2001
7	2030	Macrofauna II	PNNCP	E156	9° 47ʹ 1.00ʺ N–76° 14ʹ 12.00ʺ W	26/03/2001
8	2030	Macrofauna II	PNNCP	E156	9° 47ʹ 1.00ʺ N–76° 14ʹ 12.00ʺ W	26/03/2001
9	2032	Macrofauna II	PNNCP	E155	9° 47ʹ 12.00ʺ N–76° 13ʹ 45.00ʺ W	26/03/2001
10	2378	Macrofauna II	PNNCP	E156	9° 47ʹ 1.00ʺ N–76° 14ʹ 12.00ʺ W	01/09/2002
11	2427	MARCORAL	PNNCP	E246	9° 52ʹ 58.4ʺ N–76° 9ʹ 13.6ʺ W	30/04/2005
12	2442^a^	MARCORAL	PNNCP	D12	9° 47ʹ 5.8ʺ N–76° 13ʹ 16.1ʺ W	01/05/2005
13	CNI3495 F1^a^	ANH-ARC JAMAICA	Bajo Nuevo	EA 294	15° 57ʹ 4.39ʺ N–78° 31ʹ 16.6ʺ W	18/10/2011
14	CNI3495 F1^a^	ANH-ARC JAMAICA	Bajo Nuevo	EA 294	15° 57ʹ 4.39ʺ N–78° 31ʹ 16.6ʺ W	18/10/2011
15	3822^a^	INVEMAR–ICP	PNNCP	EA1	9° 14ʹ 57.8ʺ N–76° 27ʹ 1.8ʺ W	29/07/2012
16	3825^a^	INVEMAR–ICP	PNNCP	EA2	9° 55ʹ 36.48ʺ N–76° 8ʹ 23.8ʺ W	03/08/2012
17	3828	INVEMAR–ICP	South PNNCP	EA2	9° 16ʹ 3.54ʺ N–76° 26ʹ 26.8ʺ W	29/07/2012
18	3830^a^	INVEMAR–ICP	PNNCP	EA2	9° 56ʹ 14.1ʺ N–76° 7ʹ 26.8ʺ W	05/08/2012
19	3834	INVEMAR–ICP	PNNCP	EA2	9° 56ʹ 14.1ʺ N–76° 7ʹ 26.8ʺ W	05/08/2012
20	3835	INVEMAR–ICP	PNNCP	EA2	9° 56ʹ 14.1ʺ N–76° 7ʹ 26.8ʺ W	05/08/2012
21	3836	INVEMAR–ICP	PNNCP	EA2	9° 56ʹ 14.1ʺ N–76° 7ʹ 26.8ʺ W	05/08/2012
22	3836	INVEMAR–ICP	PNNCP	EA2	9° 56ʹ 14.1ʺ N–76° 7ʹ 26.8ʺ W	05/08/2012
23	3838^a^	INVEMAR–ICP	PNNCP	EA2	9° 56ʹ 14.1ʺ N–76° 7ʹ 26.8ʺ W	04/08/2012
24	3851^a^	INVEMAR–ICP	PNNCP	EA2	9° 56ʹ 14.1ʺ N–76° 7ʹ 26.8ʺ W	26/07/2012
25	1619^a^	PNNCP 2015	PNNCP	ST 8	9° 49ʹ 02.1ʺ N–76° 12ʹ 23.7ʺ W	15/10/2015
26	1620^a^	PNNCP 2015	PNNCP	ST 9	9° 47ʹ 05.3ʺ N–76° 13ʹ 37.5ʺ W	15/10/2015

^a^Samples with good amplification quality.

## Discussion

This study presents nine new microsatellites developed for *M. myriaster*, based on the samples collected at PNNCP (Colombia)*.* All the new microsatellites were tested in all the available samples, independently of their quality of DNA. Despite obtaining microsatellite amplification of 14 loci in the 26 samples of *M. myriaster,* with clear bands in the electrophoresis agarose get prior to the genotyping, 13 samples were excluded because some of their fragment sizes under “Fragman” software did not reach the threshold to detect them, or because the amplification was not clear to bin the fragment sizes using “MsatAllele”. Understanding that the exclusion of that data limited the study scope, the decision was taken to keep a conservative approach and eliminate false positives in the analysis.

Likewise, five microsatellite loci with low amplification in the 13 samples were excluded from the analyses. These decisions were taken to avoid possible data misinterpretation from amplification errors caused commonly by low concentration of DNA from samples kept in museums^31^, as was the case of these 13 samples with a DNA concentration below of 10 ng/μl. The low DNA concentration could create a common error during the PCR amplification, called “false homozygotes”, due to random sampling in the DNA template and deficiency amplification as a response to very low DNA quantity^32^. The literature suggests increasing the number of PCRs to reduce the occurrence of “false homozygotes”^32^; unfortunately, it was not possible to perform the additional PCRs due to lack of additional DNA material from the samples. However, it is highly likely that any of the 14 developed microsatellites, including the last five removed markers, can be amplified using samples with better quality and quantity of DNA.

The newly developed microsatellites for *M. myriaster* may be used as a tool to examine genetic variation among populations of *M. myriaster* in the Caribbean Sea once more samples from the area are available. The usefulness of these type of molecular markers in conservation studies has been reported^19^ and they have been used successfully with shallow corals^33,34^ and deep- sea corals^27,28^. In addition, microsatellites have also proven to be helpful in the design and effectiveness of MPAs, based in the data given for populations of connectivity and genetic structure derived from these markers^35^. Molecular markers have also been incorporated, as complementary tools, in the identification of species in the genus *Madracis.* Some examples include the Internal Transcribed Spacers (ITS) for genetic variation^13^, the putative mitochondrial control region-CR^36^, the ATP8 mitochondrial marker^37^, as well as the ATPS and SRP54 nuclear markers^38^. For this reason, the specificity of the new microsatellites developed for *M. myriaster* shows the potential use of these markers as a complementary taxonomic identification tool, because it was found that they amplify only in samples of this particular species and no other corals.

In this study, nine samples catalogued as *M.* cf. *myriaster* were excluded at the microsatellite standardization stage due to the non-amplification, despite the good quality and quantity of DNA. These samples could have been *M. brueggemanni* due to the morphologic similarity with *M. myriaster*^2^. However, it was not possible to confirm the identity of the samples catalogued as *M.* cf. *myriaster,* because they had the same morphological features under traditional taxonomic methods but were identified as “different” by the microsatellite standardization method. Also, the rudimentary method used to collect the samples in most of the stations in Colombia, known as trawl net sampling, causes difficulty for the correct identification of the fragments because the method breaks and mixes the colonies from different species into small fragments over a 1 km transect sampling^12,39^. These small coral fragments (less than 2 cm) are very difficult to identify under traditional taxonomic methods, as it was evident in the results, because there are not significant differences in the branch thickness, size or, the corallite diameter among samples, characters commonly used in traditional taxonomy.

Despite the obvious limitations to perform additional analyses due to the reduced number of samples currently available, additional constraints regarding the high costs and logistics to obtain deep-sea coral samples obstruct the exploration on these habitats. However, the new microsatellites presented here can be used in future analysis, considering the need to continue the exploration of marine national parks, MPAs in Colombia, and other areas in the Caribbean. Currently, the PNNCP has only been explored until 500 m depth, but there is a need to explore the deepest areas of the park, reaching 1350 m depth^40^. Personal communications with other colleagues suggest that this coral could also be present at similar depths in other Caribbean and Atlantic locations (e.g. Gulf of Mexico, Florida and Brazil)^3,41,42^. Therefore, these markers may be used in the near future as a tool to strengthen the incomplete identification of samples, to complete the base line of deep environments in MPAs, to examine the Caribbean connectivity among deep-sea habitats, and to develop comprehensive conservation management plans of MPAs in the Caribbean and Atlantic region.

## Methods

### Sampling

Fifty-three fragments of *M.* cf. *myriaster* were used for this study from the reference collection of Cnidarians at the Marine Natural History Museum of Colombia (MHNMC), part of INVEMAR. The samples belong to different exploration projects in the Colombian Caribbean Sea, from 1998 to 2015, where trawl net methodology was used at depths between 120 and 350 m, following transects of 1 km in length. After collection, all samples from the same transect were mixed and preserved in 70–90% ethanol. Table 2 shows the initial coordinates of each transect, from where samples were selected for molecular analysis (more information: https://siam.invemar.org.co/campanas-proyectos). The 53 samples were exported to Manchester Metropolitan University under the CITES permits 40,885 (25th July 2016), 41,449 (25th January2017) and 43,908 (6th May 2019).

### Analysis on *Madracis myriaster* samples

All the 53 coral fragments were originally examined to confirm the correct identification because the method used to collect the samples in Colombia, known as trawl net sampling, usually breaks the colonies into small fragments, mixing the fragments from different colonies over the 1 km transect sampling, and increasing the chance of mixing colonies of *M. myriaster* and *M. brueggemanni.* However, 18 samples originally catalogued as *M. myriaster* were eliminated due to degraded DNA (DNA extraction, PCR standardization and DNA sequencing section). Another nine samples originally catalogued as *M.* cf*. myriaster* were eliminated from the analyses due to unsuccessful amplification (DNA extraction, PCR standardization and DNA sequencing section). As a result, the identification effort was focused on the 26 samples used in the microsatellite loci characterization.

Morphological descriptions from Reyes et al., (2010), were used for the taxonomic revision for the 26 samples, taking into account the morphological similarity of *M. myriaster* with its sister species *M. brueggemanni*. *M. myriaster* or “fluted finger coral” often has branched colonies with an irregular morphology and purple, pink or orange colours. In contrast, *M. brueggemanni* forms colonies with tiny and delicate branches, giving a less complex three-dimensional structure arranged in three dimensions. *M. brueggemanni* also presents a narrow distribution across the Western Tropical Atlantic and is found between 51 and 160 m deep^2^. Twenty-four from the 26 samples were further examined due to ambiguous morphology using scanning electron microscopy-SEM (Zeiss Supra 40VP FE-SEM with an EDX detector for elemental analysis at Manchester Metropolitan University -MMU), due to the mismatch with the species descriptions above and the extremely small size of the fragment for the comparison. The images were analysed using the program Image J to quantify branch thickness (three measurements per photo), corallite diameter (two measurements per photo) and presence of spines in the sample. Afterwards, the SEM images from the 24 samples were revised with Dr Nadia Santodomingo, at the Natural History Museum of London-NHML, splitting the samples in two groups (group a with 11 samples confirmed as *M. myriaster* and, group b with 13 samples as *M.* cf. *myriaster*) in order to find morphological differences using a t-test.

### DNA extraction, PCR standardization and DNA sequencing

The following DNA extraction protocol was optimized using two samples identified as *M. myriaster* (samples codes 1619 and 1620) with the DNA tissue and blood extraction kit from Qiagen: (1) fragments of the sample (0.5—1 cm diameter) were dried at 36 °C for 10 min using a Thermomixer; (2) 180 μL of buffer AL and 20 μL of Proteinase K were added to the sample and vortexed for 20 s; (3) the samples were incubated in the Thermomixer at 55 °C for 2 h with vortex every 30 min; (4) after digestion, the fragment was removed (calcium carbonate skeleton) and 200 μL of buffer AL was added, after a brief vortex for 20 s, 200 μL cold ethanol was added; (5) all the mix was transferred to filter tubes and centrifuged at 10,000 rpm for 5 min; (6) the filter from the tube was transferred to a new collection tube, 500 μL AW1 were added and centrifuged at 10,000 rpm for 3 min; (7) the filter was transferred after the centrifuge into a new collection tube, 500 μL AW2 were added and centrifuged at 13,500 rpm for 3 min; (8) the filter tube contents were transferred into a 2 ml Eppendorf tube and 20 μL of buffer AE (pre-heated at 75 °C) were added; (9) then left for 15 min at room temperature (10) afterward centrifuged at 10,000 rpm for 1 min; (11) steps 9 and 10 were repeated and then the filter was discharged; (12) the final sample (2 ml Eppendorf tube) was stored at -20 °C. Electrophoresis was used to test the quality of the DNA extractions (53 samples) with the following conditions: (1) 0.80 g of agar were mixed with 80 ml of 1X TBE buffer solution (1% agar gels) and 0.8 μL of GEL GREEN to prepare the gels; (2), 1 μL of sample plus 2 μL of loading buffer were added into each pool. The electrophoresis chamber was run at 60 V for 60 min.

At this point, 18 samples confirmed as *M. myriaster* were excluded due to absence of DNA after the extraction. The DNA from the two samples chosen to improve the extraction protocol (1619 and 1620—good quality and quantity of DNA) were normalized to 50 ng using the Nextera® DNA sample Preparation Kit and sent to the sequencing facility at the University of Manchester—UoM (UK) to perform an Illumina MiSeq paired-end sequencing (2 × 250 bp). The sequencing data were analysed using the bioinformatics tool Palfinder on the Galaxy Centaurus Server Platform (https://palfinder.ls.manchester.ac.uk), and the primers were designed using the optional filters pipeline, with parameters for melting temperature, annealing temperature and primer length of the Type-it Microsatellite PCR QIAGEN kits^18^. From the process, 223 potentially amplifiable loci (PALs) were obtained and 36 PALs (7 tri- and 29 tetra-nucleotide motifs) with good motifs and GC contents were selected for further analysis. From the remaining 35 DNA samples, eight samples (2012, 2013, 3838, 2442, 3830, 3851,CN13495a-b; Table 2), were used to explore the amplification of the selected 36 microsatellites in 10 μL reaction mixes containing: 5 μL of Master Mix (Type-it Microsatellite PCR QIAGEN Kit), 3 μL H_2_O molecular grade, 1 μl Primer mix and 1 μl DNA (20 ng/μl). The PCRs were run (TECHNE thermocycler) under the following conditions: (1) denaturation 95 °C/5 min; (2) 32 cycles including 95 °C/30 s for denaturation, 60 °C/1.5 min for annealing, and 72 °C/30 s for elongation; (3) a final extension at 60 °C/30 min; (4) an endless holding at 4 °C. The PCR products were electrophoresed in a 0.8% agarose gel to confirm amplification of the microsatellites.

As a result of the exploration, 22 from the 36 selected loci were excluded due to irregular amplification in the samples. The remaining 14 microsatellites were redesigned with the universal tail sequences Blackett A: GCCTCCCTCGCGCCA^43^ and M13-mod B: CACTGCTTAGAGCGATGC^44^, to distinguish among amplified fragments by their fluorescent labelling dyes (6-FAM and ROX, accordingly). The new 14 microsatellites were tested in the 35 DNA samples using the program Multiplex_Manager^45^ to perform multiplexes, reducing time and cost. Each amplification run used positive and negative controls from the 8 previously tested samples. The new reaction mixes contained 5 μL of Master Mix (Type-it Microsatellite PCR QIAGEN Kit), 3 μL H_2_O molecular grade, 1 μl Primer mix (Pre_laballed Forward + Reverse + Fluorescence (6-FAM or ROX) + H_2_0 molecular grade) and 1 μl DNA (20 ng/μl). The PCR conditions were the same as described above.

Nine samples identified as *M.* cf. *myriaster* were eliminated at this point due to unsuccessful amplification. The genotyping was performed with the remaining 26 samples (Table 2). The fluorescence labelled PCR products were sent to the University of Manchester DNA Sequencing Facility (UK) and to the Core Genomics Facility at the University of Sheffield (UK), in a mix of: 9 μl of HiDi Formamide, 0,2 μl of the Liz 500 (GeneScan™ 500 LIZ®) and 0,8 μl of the PCR product (including the positive and negative controls). The products were sized in both places using the capillary electrophoresis Applied Biosystems 3730 DNA Analyser (enabling size discrimination within the range of 20 to 600 base pairs using a range of dyes). A species-specificity primer amplification test was also performed on *M. auretenra (2 samples), Montipora* sp. (2 samples) and *Antillogorgia* sp. (2 samples), to confirm no amplification on taxonomically near species.

### Data analysis

The software R was used to perform data analysis on the 26 samples, using the package ‘Fragman’^46^ to read FASTA files with different fragment sizes for each locus in each sample obtained after the genotyping. The values of each allele size were normalized using the positive control. The ‘MsatAllele’ package^47^ was employed to bin the fragment sizes (Fragman output), assigning to a set of defined alleles the closest size for each marker. Then the true allele set was determined using the known repeat length and using the histograms of observed fragment lengths before and after the binning. The package ‘Poppr’ in R^48^ was also used to assess the frequency of missing alleles across primers. Additionally, the number of clones present in the sampling was investigated by counting multi-locus genotypes throughout localities to report the observed (Ho) and expected heterozygosity (He).
